# Integrating genome-wide association study and transcriptomic profiling identifies candidate genes underlying soluble solids content in melon (*Cucumis melo* L.)

**DOI:** 10.1093/hr/uhag196

**Published:** 2026-05-13

**Authors:** Xuxu Niu, Qiong Li, Lei Cao, Yachen Liu, Junhao Qiang, Zhu Lian, Yi Xie, Qing Meng, Wenkai Yan, Panqiao Wang, Xiang Li, Wenwen Mao, Juan Hou, Lili Li, Luming Yang, Junlong Fan, Yan Guo, Zhiqiang Cheng, Chen Luo, Jianbin Hu

**Affiliations:** College of Horticulture, Henan Agricultural University, Zhengzhou 450046, China; College of Horticulture, Henan Agricultural University, Zhengzhou 450046, China; College of Horticulture, Henan Agricultural University, Zhengzhou 450046, China; College of Horticulture, Henan Agricultural University, Zhengzhou 450046, China; College of Horticulture, Henan Agricultural University, Zhengzhou 450046, China; College of Horticulture, Henan Agricultural University, Zhengzhou 450046, China; College of Horticulture, Henan Agricultural University, Zhengzhou 450046, China; College of Horticulture, Henan Agricultural University, Zhengzhou 450046, China; College of Horticulture, Henan Agricultural University, Zhengzhou 450046, China; International Joint Laboratory of Henan Horticultural Crop Biology, Zhengzhou 450046, China; College of Horticulture, Henan Agricultural University, Zhengzhou 450046, China; International Joint Laboratory of Henan Horticultural Crop Biology, Zhengzhou 450046, China; College of Horticulture, Henan Agricultural University, Zhengzhou 450046, China; International Joint Laboratory of Henan Horticultural Crop Biology, Zhengzhou 450046, China; College of Horticulture, Henan Agricultural University, Zhengzhou 450046, China; International Joint Laboratory of Henan Horticultural Crop Biology, Zhengzhou 450046, China; College of Horticulture, Henan Agricultural University, Zhengzhou 450046, China; International Joint Laboratory of Henan Horticultural Crop Biology, Zhengzhou 450046, China; College of Horticulture, Henan Agricultural University, Zhengzhou 450046, China; International Joint Laboratory of Henan Horticultural Crop Biology, Zhengzhou 450046, China; College of Horticulture, Henan Agricultural University, Zhengzhou 450046, China; International Joint Laboratory of Henan Horticultural Crop Biology, Zhengzhou 450046, China; Henan Engineering Center for Cucurbit Germplasm Enhancement and Utilization, Zhengzhou 450046, China; Kaifeng Academy of Agriculture and Forestry, Kaifeng 475000, China; Kaifeng Academy of Agriculture and Forestry, Kaifeng 475000, China; Kaifeng Academy of Agriculture and Forestry, Kaifeng 475000, China; College of Horticulture, Henan Agricultural University, Zhengzhou 450046, China; International Joint Laboratory of Henan Horticultural Crop Biology, Zhengzhou 450046, China; College of Horticulture, Henan Agricultural University, Zhengzhou 450046, China; International Joint Laboratory of Henan Horticultural Crop Biology, Zhengzhou 450046, China; Henan Engineering Center for Cucurbit Germplasm Enhancement and Utilization, Zhengzhou 450046, China

## Abstract

Soluble solids content (SSC) is a major determinant of flavor quality in melon fruit; however, the key genes regulating this trait remain poorly characterized. To elucidate the molecular basis of SSC variation, we performed genome-wide association studies (GWAS) using a diverse panel of 200 melon accessions, which detected 79 significant single nucleotide polymorphisms associated with SSC. Linkage disequilibrium decay analysis annotated 86 candidate genes within the associated genomic regions. To prioritize functionally relevant candidates, we further integrated transcriptome data from two genotypes with contrasting SSC levels, ‘San Ye Hua sugua’ (SYH; low SSC) and ‘Bai Sha Mi’ (BSM; high SSC). Comparative transcriptomic analysis revealed 18 candidate genes that were both GWAS-associated and differentially expressed between the two genotypes. Subsequent qRT-PCR validation confirmed concordant expression patterns for 14 of these candidates. Haplotype analysis demonstrated that natural variation at these loci was significantly correlated with SSC phenotypic variation across the population. Physicochemical component analysis revealed sucrose as the major driver of SSC accumulation. Five key candidate genes (*MELO3C024714.2*, *MELO3C026992.2*, *MELO3C012177.2*, *MELO3C007562.2*, and *MELO3C007563.2*) showed differential expression within the lipid metabolism-glyoxylate cycle-gluconeogenesis pathway, implicating their synergistic involvement in energy metabolism and cellular homeostasis. In conclusion, our multiomics approach identifies a robust set of candidate genes modulating melon SSC, providing mechanistic insights into fruit flavor formation and offering valuable genetic targets for marker-assisted or genomics-informed breeding of high-quality melons.

## Introduction

Melon (*Cucumis melo* L.; 2*n* = 2*x* = 24) is a globally cultivated and economically important fruit vegetable [1]. According to FAOSTAT (2023; https://www.fao.org/faostat/en/#data/QCL), global melon production reached approximately 29.5 billion tons. Beyond yield, fruit quality is a major focus for both breeders and consumers. Soluble solids content (SSC), encompassing substances such as soluble sugars, organic acids, and vitamins, serves as a critical indicator for evaluating fruit flavor quality [2]. Notably, soluble sugars alone typically account for 55%–65% of total SSC [3, 4], with high SSC reflecting either elevated sugar and reduced acid levels or vice versa. The concentrations of fructose, sucrose, maltitol, erythritol, and maltose with SSC were clustered by Sauvage *et al.* [5] through correlation analysis. Therefore, SSC is a key quality trait intimately linked to melon fruit flavor and remains a primary phenotypic target for dissecting its underlying genetic regulators.

Fruit SSC is a quantitative trait governed by multiple genes and susceptible to environmental influences [6]. Elucidating the key loci or genes controlling this trait is pivotal for the improvement of edible crops, with cucurbit crops benefiting significantly in this field. Current research has employed diverse approaches to identify SSC-associated loci in cucurbits. For instance, an SSC regulatory gene *BhSSC2.1* was mined in wax gourd via QTL mapping based on a high-density genetic mapping combined with bulked segregant analysis (BSA)-seq [7]. In winter squash, five SSC-related QTLs (*ssc3.1*, ssc4.1, *ssc11.1*, *ssc13.1*, and *ssc17.1*) were detected using an InDel marker-anchored genetic map [8]. For melon, an early study constructed a linkage map with 96 cleaved amplified polymorphic sequence (CAPS) markers from an F_2_ population derived from the cross MR-1 × Topmark, leading to detecting eight QTLs related to brix content: five detected for the fruit periphery (chromosomes 5, 7, 10, 11, and 12) and three for the central part (chromosomes 1, 11, and 12) [9]. Subsequently, Xu *et al.* [10] employed 104 SSR markers for association mapping and detected 16 SSR loci associated with the fruit SSC across 10 chromosomes, seven of which were coincided with the previously reported QTLs [11–14]. In another study, Amanullah *et al.* [15] used an F_2_ mapping population of 148 individuals genotyped with 163 single nucleotide polymorphism (SNP)–CAPS markers, detecting six SSC-associated QTLs (*Brix1.1*, *Brix1.2*, *Brix4.1*, *Brix6.1*, *Brix9.1*, and *Brix12.1*) on chromosomes 1, 4, 6, 9, and 12, respectively. Meanwhile, Wang *et al.* [16] identified 11 SNPs linked to SSC on chromosomes 1, 2, 3, and 4 in a core collection of 383 melon accessions. Despite these advances, genetic dissection of SSC in melon has largely confined to the initial identification of QTLs, while fine mapping and subsequent elucidation of causal genes lagging considerably.

Genome-wide association study (GWAS) has emerged as an efficient approach for identifying QTL and candidate genes, and has been widely applied in crops and horticultural plants [16, 17]. By analyzing associations between genetic variations and phenotypic traits in natural populations, GWAS facilitates the localization of significant SNP loci and subsequent candidate gene discovery. For instance, in tomato, Wang *et al.* [18] identified a novel QTL, *STP1*, which encodes a sugar transporter protein strongly correlated with SSC levels, using GWAS across 481 tomato accessions. Similarly, McLeod *et al.* [19] detected multiple candidate genes in pepper associated with fruit flavor (8 genes), fruit size and shape (28 genes), fruit color (32 genes), plant growth and earliness (15 genes), and plant productivity (14 genes). GWAS has also been extensively applied to cucurbit crops (including cucumber, watermelon, and melon) to uncover genetic loci underlying various agronomic traits. Guo *et al.* [20], for instance, identified 44 association signals for traits including SSC, fruit color, fruit shape, stripe pattern, and seed color across 414 watermelon accessions, and further validated that the *ClBt* gene significantly reduces sugar content via gene editing. In cucumber, Bo *et al.* [21] pinpointed *Csa5G021320* as a candidate gene regulating flesh color, while Lee *et al.* [22] identified *Csa5G471070* and *Csa5G453160* as powdery mildew resistance-associated genes. In melon, however, most GWAS efforts have focused on leaf and fruit morphological traits. For example, Zhao *et al.* [23] detected the *CmPH* gene within association signals from a GWAS for flesh acidity, agreeing with the previous findings by Cohen *et al.* [24]. By combining GWAS and domestication analysis on 297 melon accessions, Liu *et al.* [25] identified *CmCLV3*, a gene with pleiotropic effects on carpel number and fruit shape. More recently, Yan *et al.* [26] integrated QTL mapping, GWAS, and expression analysis to identified five SSC-related candidate genes. Nevertheless, systematic investigations into melon fruit quality traits, particularly SSC, remain relatively limited.

In this study, we first performed GWAS on a natural population of 200 melon accessions phenotyped for SSC across six environments, identifying a series of SSC-associated SNPs. Candidate genes were then annotated based on linkage disequilibrium (LD) decay analysis. We then performed RNA sequencing (RNA-Seq) on developing fruits from two accessions with contrasting SSC levels, revealing numerous differentially expressed genes (DEGs). By integrating the GWAS and transcriptomic data, we identified overlapping candidate genes, which were further validated through expression pattern and haplotype analysis, ultimately pinpointing key candidate genes associated with SSC in melon fruit. Collectively, this work provides a molecular framework for understanding the genetic basis of melon fruit flavor quality and offers valuable theoretical insights and genetic resources for the targeted breeding of high-flavor melon cultivars.

## Results

### Phenotypic variation and heritability analysis of SSC in a natural melon population

SSC was measured at fruit maturity in a natural melon population across six environments. The phenotypic distribution of SSC approximated normality and exhibited a high coefficient of variation, indicating substantial phenotypic diversity (Fig. 1A). Broad-sense heritability (*H^2^*) for SSC was estimated at 0.95 (Supplementary Tables S1 and S2), underscoring the predominant influence of genetic factors on this trait. Strong positive correlations were observed among all pairwise environment combinations as well as between individual environments and Blup values, with Pearson correlation coefficients ranging from 0.802 to 0.947 (Fig. 1C). Phenotypic comparisons showed no significant difference in SSC between wild accessions and *C. melo* ssp. *melo*, whereas significant differences were detected between wild accessions and *C. melo* ssp*. agrestis*, as well as between the two subspecies (*melo* vs. *agrestis*) (Fig. 1B)*.* These findings suggested that SSC had likely been modified by artificial selection during evolution. Moreover, the distinct SSC profiles between *melo* and *agrestis* support the hypothesis of independent domestication trajectories for these two subspecies, a notion consistent with prior genomic evidence identifying distinct sets of domestication sweeps in each lineage [23].

**Figure 1:**
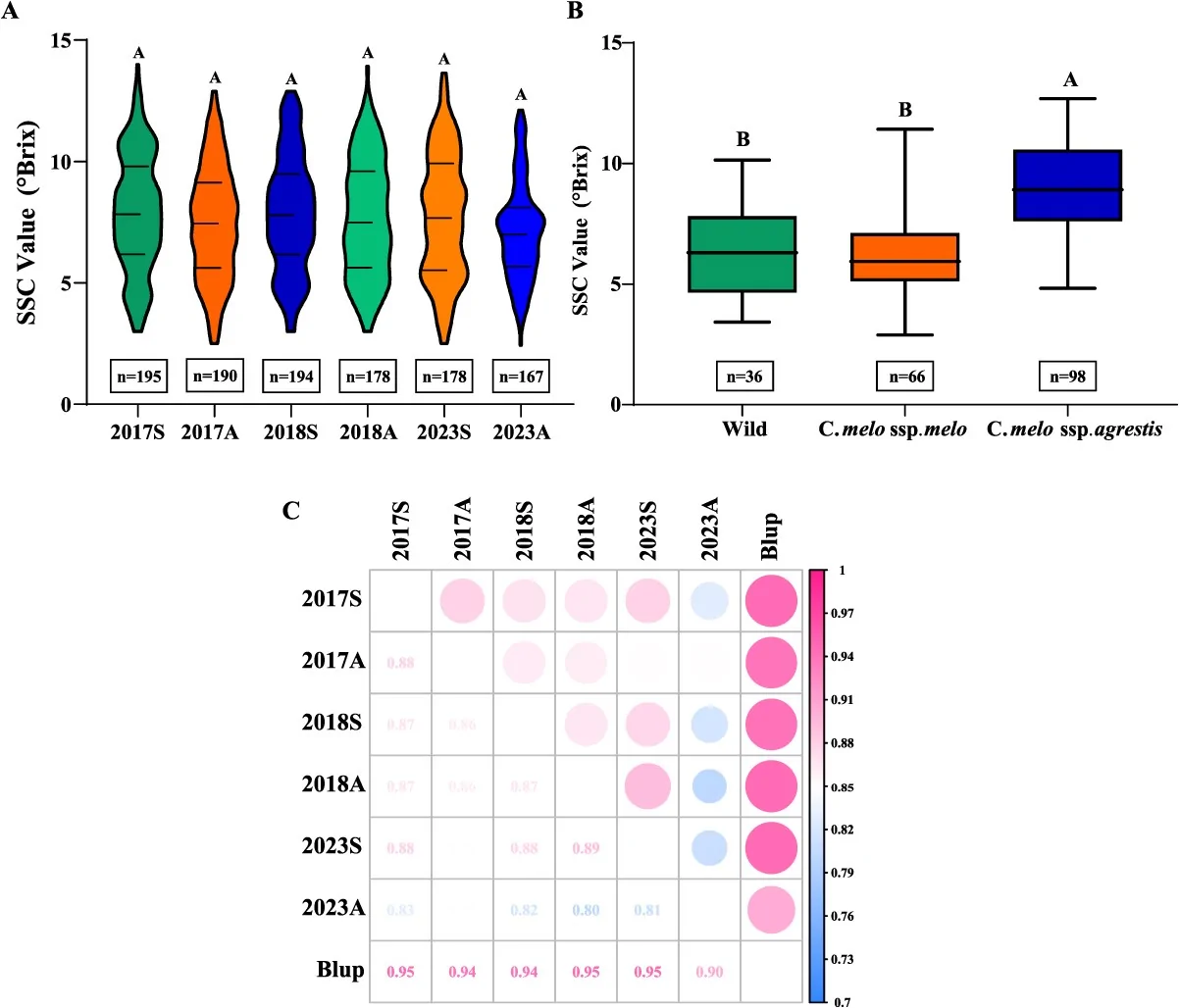
Phenotypic variation in soluble solids content across six environments. (A) SSC values of melon accessions were measured in six independent field environments and used for GWAS. *n* indicates the number of accessions per environment. Detailed accession information and raw SSC data are provided in Supplementary Table S1. (B) Phenotypic distribution of SSC among melon subspecies (Wild, C. *melo* ssp. *melo*, and C. *melo* ssp. *agrestis*). Values represent mean SSC across all accessions within each group. (C) Pearson correlation coefficients of SSC values across the six environments and best linear unbiased prediction (Blup) in the association panel. The correlation matrix was generated using SPSS. The color scale is shown on the right. Different uppercase letters in (A) and (B) represent statistically significant differences at *P* < 0.01 (one-way ANOVA followed by Tukey’s HSD test)

### Genome-wide association study identifies loci associated with SSC

To dissect the genetic architecture underlying SSC, we performed a GWAS on 200 diverse melon accessions. Using both mixed linear model (MLM) and compressed mixed linear model (CMLM) models, we identified 79 genome-wide significant SNPs (*P* < 3.94 × 10^−7^) (Supplementary Table S3). Manhattan plots highlighted two robust QTLs, *qSSC2.2* and *qSSC8.1*, which were consistently detected across multiple environments and models, underscoring their potential as major-effect QTLs (Fig. 2). Most QTLs, however, exhibited environment- or model-specific detection. For example, *qSSC2.1* was identified only in the Blup dataset under the MLM model, while *qSSC2.3* and *qSSC2.4* were specific to the 2023S environment (MLM), and *qSSC4.1*, *qSSC5.1*, *qSSC10.1*, *qSSC10.2*, and *qSSC11.1* were uniquely detected in the 2018S environment under the CMLM model. Despite their conditional detection, these loci represent plausible candidate regions contributing to SSC plasticity and may harbor valuable allelic variation. Based on the physical positions of the significant SNPs and LD decay patterns, we delineated 49 LD blocks encompassing 49 lead SNPs. The SNP in each block was extracted to predict linked genes, resulting in the identification of 86 candidate genes potentially related to SSC (Supplementary Table S4).

**Figure 2:**
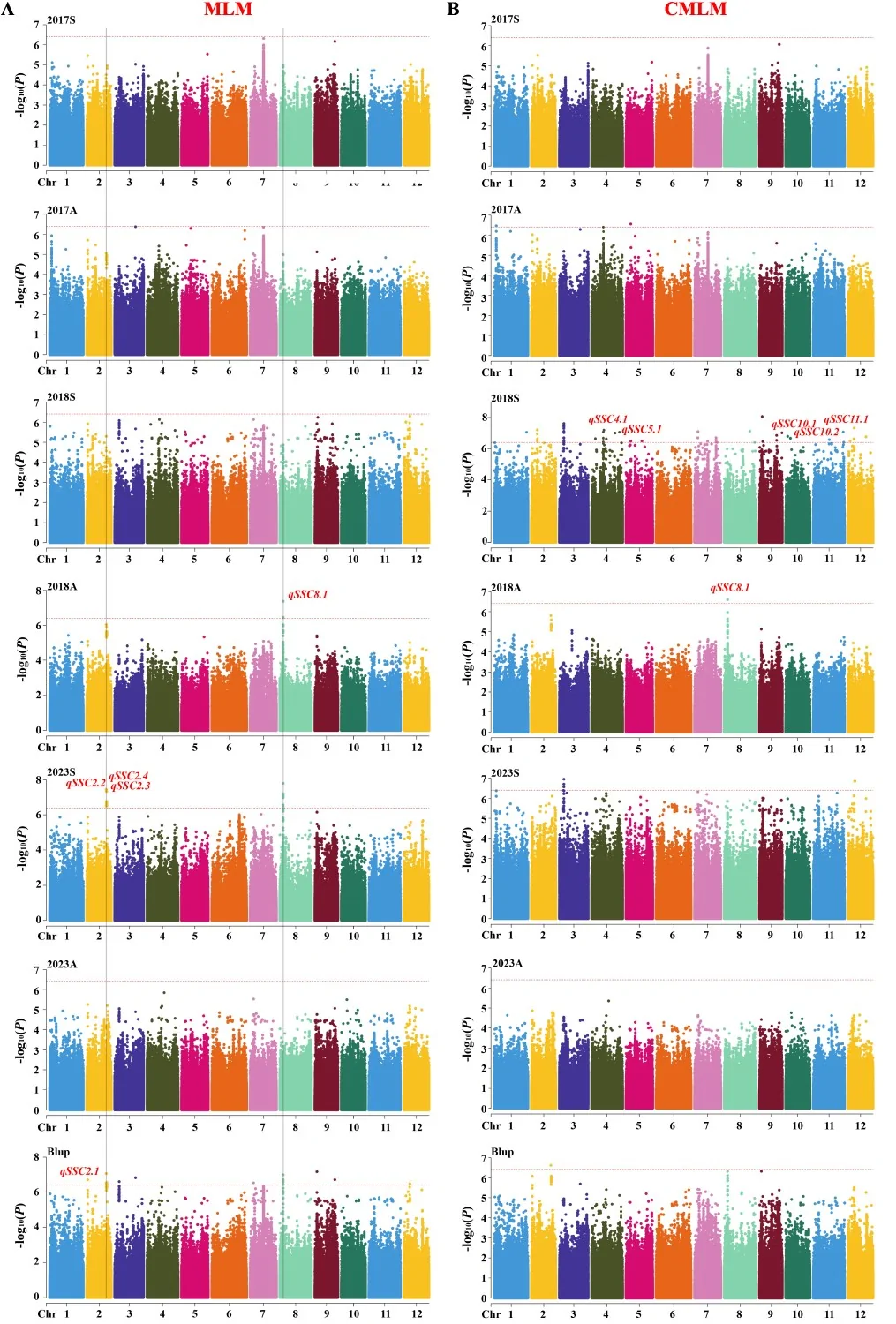
Manhattan plots from GWAS of SSC in six environments using MLM. (A) and CMLM (B) models. The *Y*-axis represents –log_10_(*P*) obtained from the GWAS results. The red horizontal lines indicate the genome-wide significance threshold of 6.40. The black vertical lines denote *qSSC2.2* and *qSSC8.1*. *qSSC2.2* was detected in 2023S and Blup under MLM model. *qSSC8.1* was detected in 2018A, 2023S, and Blup under MLM model, as well as in 2018A under CMLM model. Other peaks represent QTLs identified in individual environments or models.

### Linking SSC dynamics with transcriptomics identifies DEGs

To characterize the temporal dynamics of SSC accumulation, we selected two accessions with contrasting SSC levels, San Ye Hua sugua (SYH) (low SSC) and Bai Sha Mi **(**BSM) (high SSC). Throughout fruit development, SSC remained relatively stable in SYH. In contrast, BSM exhibited significantly higher SSC at both 20 and 30 days after pollination (DAP), with a continuous increase initiating around 20 DAP (Fig. 3B). This rapid accumulation phase is consistent with previous reports on sugar metabolism during melon ripening [27–29], suggesting that the period around 20 DAP represents a critical developmental window for regulatory events governing SSC in melon.

**Figure 3:**
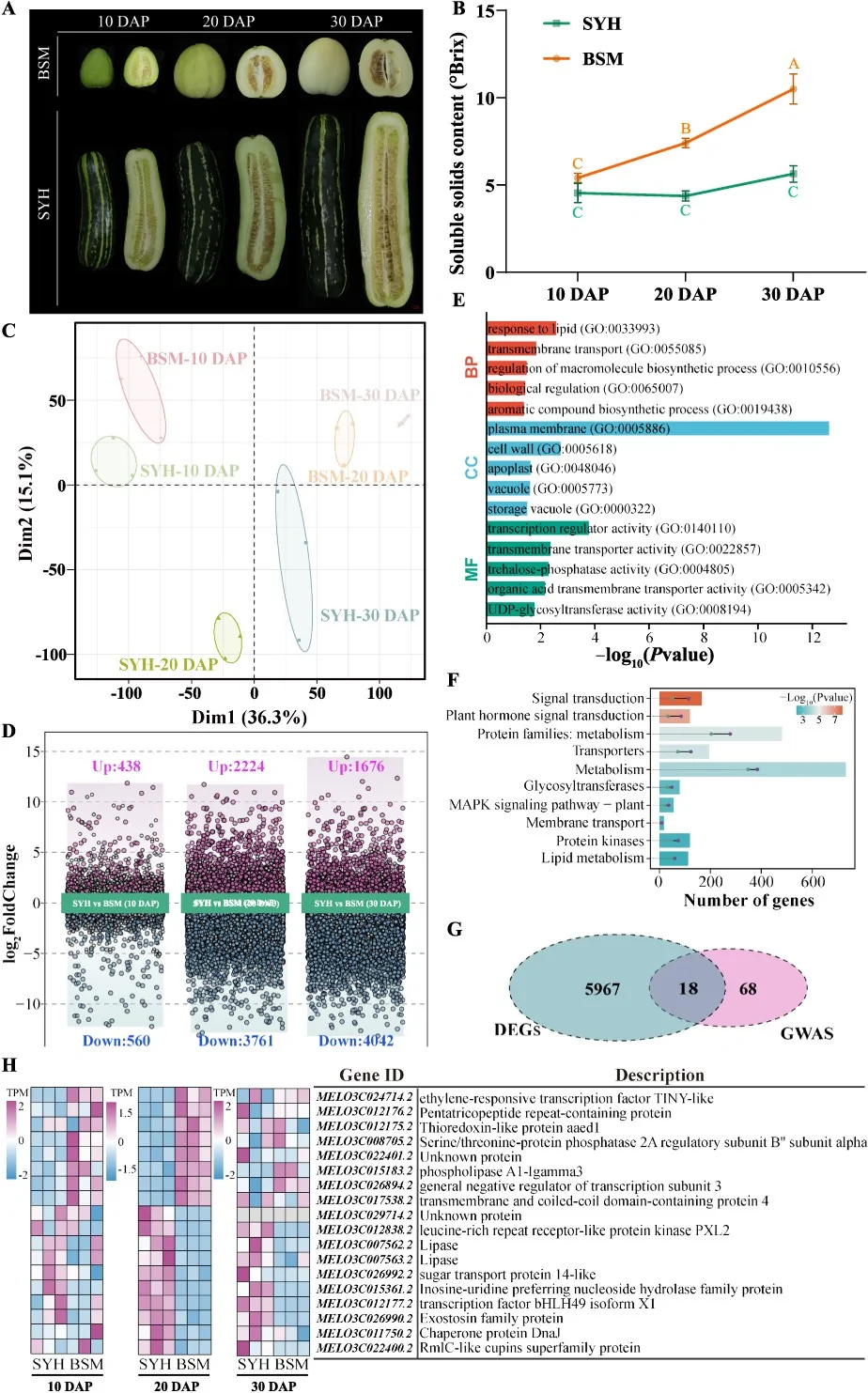
Phenotypic comparison and transcriptomic profiling of BSM and SYH fruits at different developmental stages. (A) Fruit morphology of BSM and SYH at 10, 20, and 30 DAP. Scale bars: 1 cm. (B) SSC dynamics of melon fruits across different developmental stages. Data are means ± SD (*n* = 3 biological replicates). Uppercase letters represent significant differences at *P* < 0.01 (one-way ANOVA followed by Tukey’s HSD test). (C) PCA of transcriptome profiles from SYH and BSM fruit samples at 10, 20, and 30 DAP (*n* = 3 biological replicates per sample). (D) Number of differentially expressed genes (DEGs) between SYH and BSM fruits at each stage. DEGs were selected with adjusted *P* (padj) < 0.05 and |log_2_FC| > 1. (E and F) GO (E) and KEGG (F) enrichment analyses of DEGs between SYH and BSM fruits at 20 DAP. (G) Venn diagram showing the overlap between DEGs at 20 DAP and GWAS-identified candidate genes. The overlapping regions indicate shared genes. (H) Heatmap of expression levels for candidate genes in SYH and BSM fruits across developmental stages.

Subsequently, transcriptome analysis of SYH and BSM fruits at three key developmental stages (10, 20, and 30 DAP) was conducted. Principal component analysis (PCA) revealed clear separation between the two genotypes across all stages, with high biological reproducibility. The first two principal components (Dim1: 36.3%; Dim2: 15.1%) cumulatively explained 51.4% of total transcriptomic variation (Fig. 3C). A total of 998 (438 up-regulated and 560 down-regulated), 5985 (2224 up-regulated and 3761 down-regulated), and 5718 (1676 up-regulated and 4042 down-regulated) DEGs were identified at 10, 20, and 30 DAP, respectively (Fig. 3D; Supplementary Table S5). Given the sharp rise in SSC in BSM beginning at 20 DAP, along with supporting prior physiological evidence [27–29], we prioritized DEGs at this stage for subsequent functional analysis.

Gene ontology (GO) enrichment of 20-DAP DEGs revealed significant overrepresentation in biological processes related to lipid response (GO:0033993) and transmembrane transport (GO:0055085) (Supplementary Table S6), suggesting potential disruptions in soluble solids synthesis or decomposition processes, along with alterations in lipid response and transmembrane transport. Cellular component analysis highlighted enrichments in plasma membrane (GO:0005886) and cell wall (GO:0005618), suggesting that intercellular transport and apoplastic dynamics may influence SSC. Molecular function terms included transcription regulator activity (GO:0140110), transmembrane transporter activity (GO:0022857), and trehalose-phosphatase activity (GO:0004805) (Fig. 3E). Given the significant difference in SSC between SYH and BSM, we speculated that these DEGs might play major roles in energy metabolism and homeostasis in melon fruit.

KEGG pathway enrichment analysis further revealed that the DEGs were primarily enriched in core metabolic pathways, transport pathways, and signal transduction pathways (Fig. 3F). Together, these results pointed to a complex regulatory framework involving energy metabolism, solute transport, and transcriptional regulation that likely underpins natural variation in melon SSC.

### Integration of GWAS and transcriptomics prioritizes candidate genes for SSC

To narrow the list of candidate genes, we intersected GWAS-derived loci with DEGs from the transcriptome. This integrative approach yielded 18 high-confidence candidate genes located within GWAS-defined LD blocks and differentially expressed between SYH and BSM (Fig. 3G). These genes encode proteins with diverse functional annotations, including transcription factors such as ETHYLENE RESPONSIVE FACTOR (ERF) family protein (*MELO3C024714.2*); basic Helix–Loop–Helix (bHLH) family protein (*MELO3C012177.2* and *MELO3C026894.2*); a sugar transporter (*MELO3C026992.2*); a glycosyltransferase (*MELO3C026990.2*); hydrolases (*MELO3C015183.2*, *MELO3C007562.2*, *MELO3C007563.2*, *MELO3C015361.2*, and *MELO3C017538.2*); a protein kinase (*MELO3C012838.2*); a phosphatase (*MELO3C008705.2*); a tandem amino acid motif superfamily protein (*MELO3C012176.2*); a redox-regulatory protein (*MELO3C012175.2*); a chaperone protein (*MELO3C011750.2*); an RmlC-like cupins superfamily protein (*MELO3C022400.2*); and two unknown proteins. Expression profiling at 20 DAP revealed that ten of these candidates were more highly expressed in SYH, while eight showed elevated expression in BSM (Fig. 3H), providing a foundation for functional validation of their roles in modulating SSC.

### Validation of expression patterns for key candidate genes

To corroborate the transcriptomic findings, we performed qRT-PCR to assess the relative expression levels of the 18 candidate genes (Fig. 4; Supplementary Fig. S1). Fourteen of these candidate genes exhibited expression patterns consistent with the RNA-Seq dataset [30]. In contrast, three genes (*MELO3C026894.2*, *MELO3C012176.2*, and *MELO3C017538.2*) showed no significant differential expression between two genotypes, whereas one gene (*MELO3C015183.2*) exhibited trends opposite to the transcriptome data. Given the complexity of gene structure and the differences in platform sensitivity [30–32], the four candidate genes exhibiting discordance between qRT-PCR and RNA-Seq data were temporarily excluded from the core validation set. Among the validated genes, ten genes (*MELO3C007562.2*, *MELO3C012177.2*, *MELO3C026990.2*, *MELO3C026992.2*, *MELO3C029714.2*, *MELO3C012838.2*, *MELO3C015361.2*, *MELO3C011750.2*, *MELO3C022400.2*, and *MELO3C007563.2*) showed significantly higher expression in SYH, while four genes (*MELO3C024714.2*, *MELO3C012175.2*, *MELO3C008705.2*, and *MELO3C022401.2*) were expressed at significantly lower levels in SYH. Detailed information for the 14 key candidate genes was summarized in Table 1, with comprehensive annotations provided in Supplementary Table S9. Notably, the key candidate genes identified in *qSSC2.1*, *qSSC4.1*, *qSSC5.1*, *qSSC8.1*, and *qSSC10.1* co-localized with the previously reported SSC-related QTLs [12, 33], further supporting our findings.

**Figure 4:**
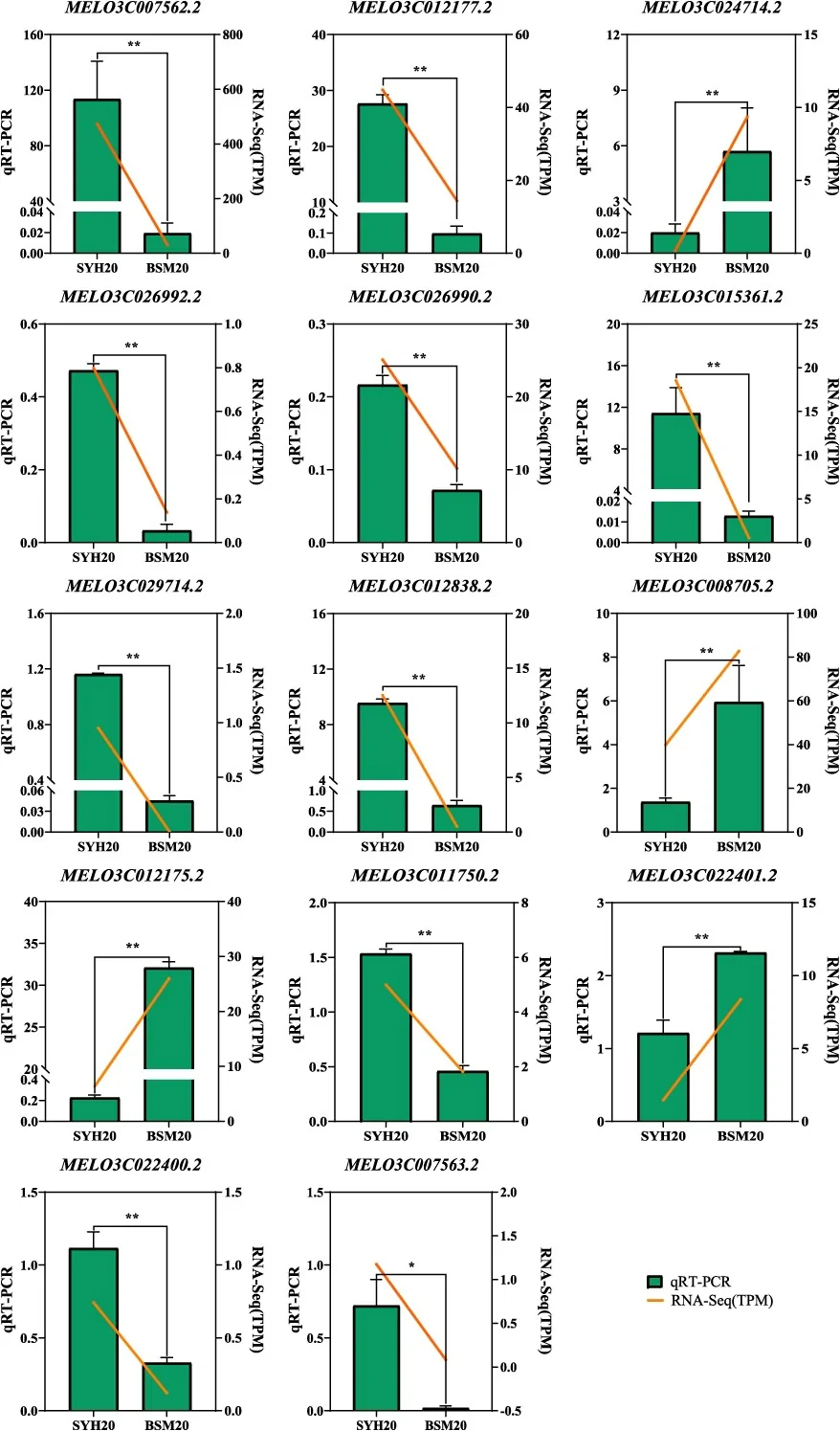
Expression validation of 14 key candidate genes between SYH and BSM fruits at 20 DAP. Left and right *Y*-axis represent qRT-PCR and RNA-Seq data, respectively. Bar plots represent the relative expression levels determined by qRT-PCR (means ± SD, *n* = 3). Asterisks indicate significant differences (^**^P < 0.01, ^*^P < 0.05; Student’s *t*-test). Superimposed lines with dots represent the TPM values obtained from RNA-Seq data.

**Table 1:** Key candidate genes associated with SSC in melon fruit.

**QTL name**	**Lead SNP**	**Ref**	**Alt**	***P*-value**	**Model**	**Environment**	**LDBlock Interval**	**Gene Id**	**Description**	**References**
*qSSC2.1*	chr02_ 1 064 505	A	C	1.96E-07	MLM	Blup	1 050 511.. 1 067 612	*MELO3C015361.2*	Inosine-uridine preferring nucleoside hydrolase family protein	Argyris *et al.* [32]
*qSSC2.3*	chr02_ 21 596 049	T	C	2.20E-07	MLM	2023S	21 596 049.. 21 603 577	*MELO3C029714.2*	Unknown protein	
				3.01E-07	MLM	Blup				
	chr02_ 21 645 912	G	A	2.91E-07	MLM	2023S	21 625 033.. 21 711 221	*MELO3C024714.2*	Ethylene-responsive transcription factor TINY-like	
	chr02_ 21 790 857	G	A	4.65E-08	MLM	2023S	21 781 760.. 21 834 651	*MELO3C026990.2*	Exostosin family protein	
								*MELO3C026992.2*	Sugar transport protein 14-like	
*qSSC4.1*	chr04_ 12 966 806	A	C	8.37E-08	CMLM	2018S	12 859 669.. 12 968 873	*MELO3C012838.2*	Leucine-rich repeat receptor-like protein kinase PXL2	Argyris *et al.* [32]
*qSSC5.1*	chr05_ 17 537 573	C	T	3.19E-07	CMLM	2018S	17 524 648.. 17 631 687	*MELO3C008705.2*	Serine/threonine-protein phosphatase 2A regulatory subunit B″ subunit alpha	Eduardo *et al.* [11]
*qSSC8.1*	chr08_ 3 682 159	C	G	4.37E-08	MLM	2018A	3 678 089.. 3 683 552	*MELO3C007562.2*	Lipase	Argyris *et al.* [32]
				1.63E-08	MLM	2023S		*MELO3C007563.2*	Lipase	
				1.02E-07	MLM	Blup				
				2.54E-07	CMLM	2018A				
*qSSC10.1*	chr10_ 2 204 733	T	C	1.63E-07	CMLM	2018S	2 190 592.. 2 222 628	*MELO3C012177.2*	Transcription factor bHLH49 isoform X1	Eduardo *et al.* [11]
								*MELO3C012175.2*	Thioredoxin-like protein aaed1	
*qSSC10.2*	chr10_ 5 210 318	C	T	2.15E-07	CMLM	2018S	5 206 196.. 5 212 726	*MELO3C011750.2*	Chaperone protein DnaJ	
*qSSC11.1*	chr11_ 33 432 772	C	G	3.86E-07	CMLM	2018S	33 421 160.. 33 433 796	*MELO3C022400.2*	RmlC-like cupins superfamily protein	
								*MELO3C022401.2*	Unknown protein	

### Haplotype analysis reveals natural variants associated with SSC

To assess the potential contribution of natural genetic variation in these candidate genes to SSC in melon fruit, we conducted haplotype-based association analyses. All the 14 key candidate genes exhibited significant haplotype-SSC associations (Fig. 5; Supplementary Fig. S2), with strong LD observed among multiple SNPs within each gene region, indicating that functional variants likely reside in coding or regulatory sequences of these loci.

**Figure 5:**
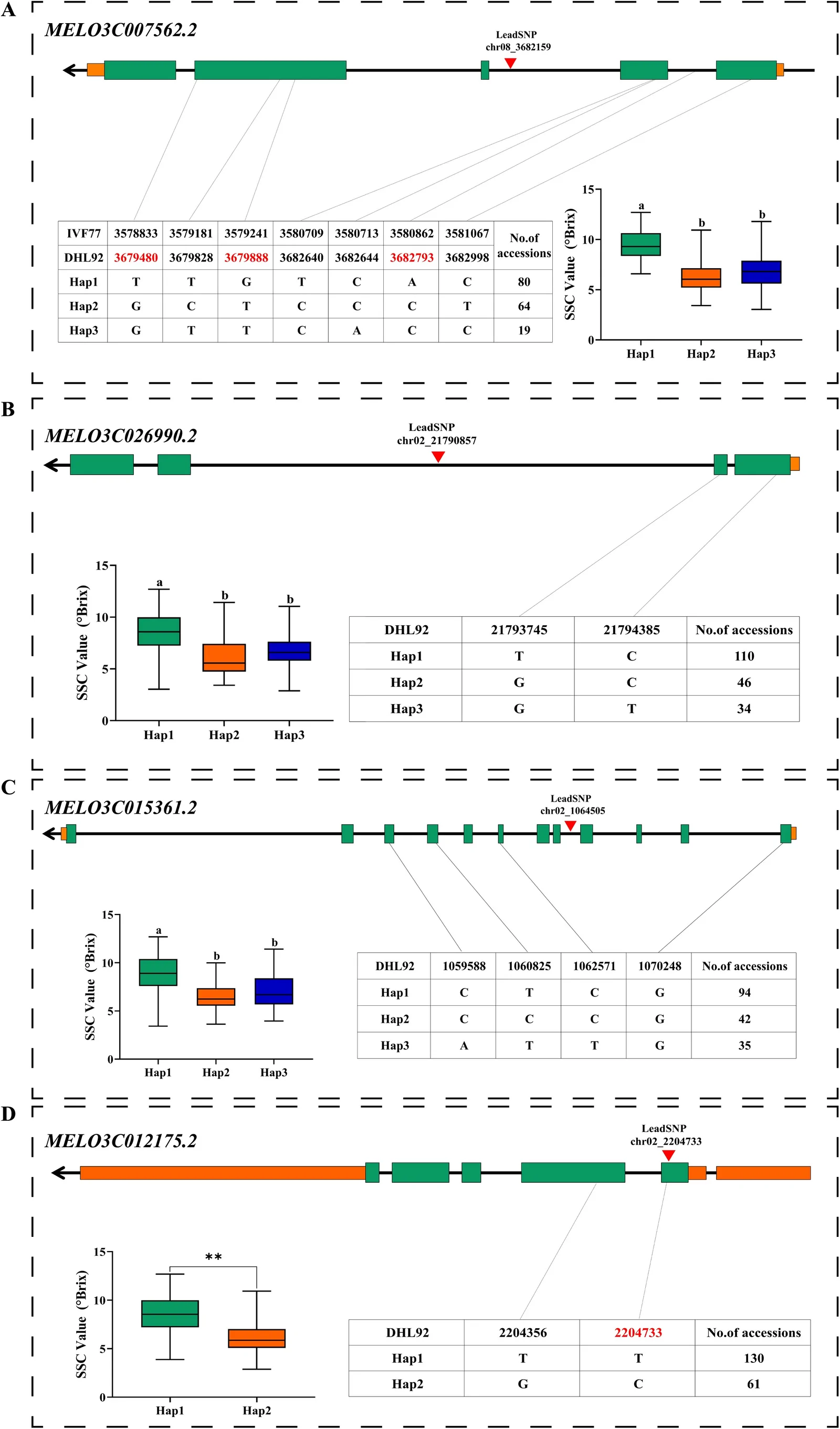
Nonsynonymous mutation and haplotype analysis of key candidate genes associated SSC. (A–D) Gene structures and haplotype effects for *MELO3C007562.2* (A), *MELO3C026990.2* (B), *MELO3C015361.2* (C), and *MELO3C012175.2* (D). Green boxes represent exons, lines represent introns, orange boxes represent the 5′UTRs and 3′UTRs, arrows indicate transcriptional direction, and red inverted triangles mark the location of lead SNPs from GWAS. Bar graphs show haplotype-associated phenotypic differences in SSC. Lowercase letters indicate statistically significant differences at *P* < 0.05 (one-way ANOVA followed by Tukey’s HSD test). ^**^, *P* < 0.01 (Student’s *t*-test). Red font in nonsynonymous variant tables indicates significant SNPs identified by GWAS.

For example, the lead SNP chr08_3 682 159, detected repeatedly across both the 2018A and 2023S, was located within an LD block that also contained three other significant SNPs chr08_3679480, chr08_3679888, and chr08_3682793. This block encompassed the gene *MELO3C007562.2*. Notably, coordinates for these SNPs differ between genome assemblies: in the IVF77 reference genome, they correspond to chr08_3578833, chr08_3579241, and chr08_3580862, respectively. Among them, chr08_3679480 and chr08_3679888, along with four additional SNPs, resulted in nonsynonymous mutations, while chr08_3682793 caused a frameshift mutation leading to a premature stop codon in *MELO3C007562.2*. Accessions were grouped into three haplotypes based on these variants, with those carrying haplotype 1 showing significantly higher SSC than those with haplotypes 2 or 3 (Fig. 5A).

Similarly, the lead SNP chr02_21 790 857, situated in an intronic region of *MELO3C026990.2*, defined a haplotype block that also included additional SNP. This gene was also categorized into three haplotypes, with haplotype 1 exhibiting significantly higher SSC relative to haplotypes 2 and 3 (Fig. 5B). Another lead SNP, chr02_1 064 505, resided within in an intron of *MELO3C015361.2* and co-segregated with three neighboring SNPs in a single LD block. Together with another SNP from a distinct block, these variants collectively generated a nonsynonymous substitution, thereby dividing *MELO3C015361.2* into three haplotypes (Fig. 5C). In *MELO3C012175.2*, two nonsynonymous mutations were identified, one of which (chr10_2 204 733) served as the lead SNP. These variants defined two haplotypes, with accessions harboring haplotype 1 displaying significantly higher SSC than those with haplotype 2 (Fig. 5D). Collectively, these haplotype–phenotype associations provide further genetic evidence that natural allelic variation in the identified candidate genes contributes to the regulation of SSC in melon.

### The lipid metabolism-glyoxylate cycle-gluconeogenesis pathway uncovers an antagonistic relationship between lipid catabolism and sugar accumulation in melon fruit

KEGG pathway enrichment analysis led us to hypothesize that, during melon fruit development, a coordinated regulatory network-comprising enzymes, transporters, and transcription factors—orchestrates energy metabolism and maintains cellular homeostasis. To illustrate this interplay, we constructed a simplified schematic of the lipid metabolism–glyoxylate cycle–gluconeogenesis (LGG) metabolic network and mapped the DEGs onto it (Fig. 6). The LGG network revealed that the majority of enzymes and transporters were upregulated in BSM (high-SSC genotype) compared to SYH (low-SSC genotype), underscoring their pivotal role in integrating carbohydrate and lipid metabolism. A total of 73 DEGs were embedded within this pathway, participating in lipid breakdown, glyoxylate cycle maintenance, and gluconeogenesis.

**Figure 6:**
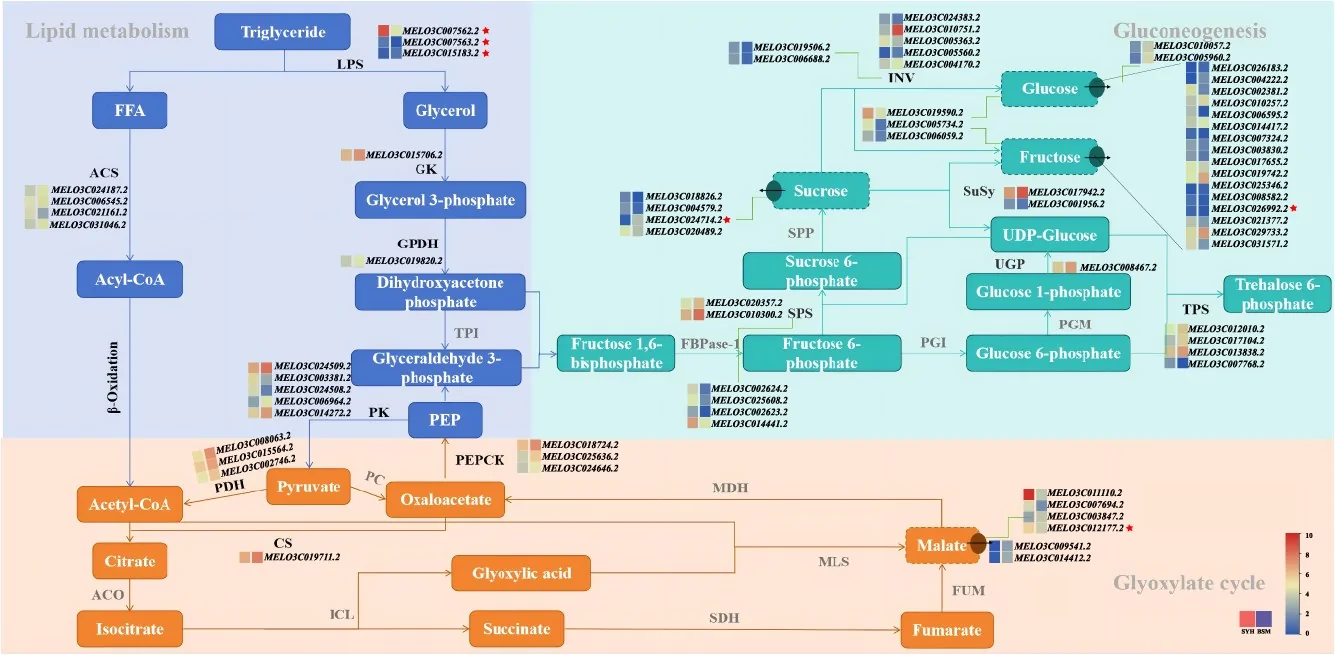
Integrated view of the LGG pathway in melon fruit. The light purple block represents the lipid metabolism pathway, the light orange block represents the glyoxylate cycle pathway, and the light green block represents the gluconeogenesis pathway. Heatmap indicates average TPM values for DEGs involved in the corresponding pathways in SYH (left) and BSM (right) fruits at 20 DAP. Red asterisks indicate GWAS-identified candidate genes. On the pathway, rounded rectangles represent metabolites, arrows represent reaction directions, letters besides the arrows represent enzymes, and ellipses with black arrows represent transporters. Blue-green broken lines represent the transcription factors that regulate enzymes or transporters. FFA, free fatty acid; Acyl-CoA, acyl-coenzyme A; PEP, Phosphoenolpyruvate; LPS, lipase; ACS, acyl-coenzyme A synthetase; ACO, aconitase; ICL, isocitrate lyase; SDH, succinate dehydrogenase; FUM, fumarase; MLS, Malate synthase; MDH, malate dehydrogenase; CS, citrate synthase; PDH, pyruvate dehydrogenase; PC, pyruvate carboxylase; PK, pyruvate kinase; GK, glycerol kinase; GPDH, glycerol-3-phosphate dehydrogenase; TPI, triose phosphate isomerase; PEPCK, phosphoenolpyruvate carboxykinase; FBPase-1, fructose-1,6-bisphosphatase; SPS, Sucrose-phosphate synthase; SPP, Sucrose-phosphate phosphatase; INV, invertase; SuSy, sucrose synthetase; PGI, phosphoglucoseisomerase; PGM, phosphoglucomutase; UGP, UDP-glucose pyrophosphorylase; TPS, trehalose-6-phosphate synthase.

Among the 14 key candidate genes identified through our integrated GWAS and transcriptomic analyses, five genes (*MELO3C024714.2*, and *MELO3C026992.2*, *MELO3C012177.2*, *MELO3C007562.2*, and *MELO3C07563.2*) were localized to the LGG pathway. Another candidate gene (*MELO3C015183.2*) was also mapped to this pathway. Notably, *MELO3C007562.2*, which encodes a putative lipase likely initiating lipid catabolism, exhibited the strongest differential expression among all 73 DEGs. Surprisingly, however, its transcript levels were significantly higher in SYH (low SSC) than in BSM (high SSC). This inverse correlation between *MELO3C007562.2* expression and SSC accumulation suggested that elevated lipolytic activity might contribute to the maintenance of a low-sugar phenotype. Rather than promoting sugar biosynthesis, robust lipid breakdown in SYH might redirect carbon resources toward alternative metabolic pathways-such as the production of volatile compounds or energy generation, thereby potentially constraining sucrose accumulation during fruit development. Additionally, the bHLH transcription factor *MELO3C012177.2* was significantly upregulated in SYH. Based on its expression pattern, we proposed that *MELO3C012177.2* acted as a negative regulator that suppressed metabolic or developmental programs necessary for the transition from a low-SSC to high-flavor-quality fruit state, thereby reinforcing the low-sugar phenotype.

### Correlating physicochemical profiles with candidate gene expression

To deconstruct the complex SSC trait into specific metabolic components and precisely evaluate the functions of candidate genes, we quantified soluble sugars (sucrose, glucose, and fructose), organic acids (malate and citrate), and fatty acids (palmitic acid, stearic acid, linoleic acid, and oleic acid) in SYH and BSM fruits (Fig. 7A). At 30 DAP, sucrose content in BSM accounted for 65% of the total soluble sugar content, indicating that sucrose is the primary sugar component driving the elevated SSC in BSM. Regarding organic acids, malate content decreased sharply from 10 to 20 DAP in both accessions, with the most prominent change observed in BSM, while citrate content increased rapidly during the same period—a trend most pronounced in BSM. By 30 DAP, citrate became the predominant organic acid in both accessions, however, its content was significantly lower than that of soluble sugars, suggesting that it may play an auxiliary role in modulating fruit flavor. Compared to 10 DAP, the fatty acid content in both SYH and BSM decreased significantly at 20 DAP, especially in BSM. This time window coincides with the period of sucrose accumulation and malate reduction, suggesting that fatty acid degradation may provide carbon skeletons and energy for gluconeogenesis and the glyoxylate cycle. Correlation analysis revealed that SSC was highly positively correlated with sucrose content (*R*^2^ = 0.8986, *P* < 0.001) (Fig. 7C), demonstrating that sucrose accumulation is the primary driver of SSC variation and highlighting the importance of elucidating the regulatory mechanisms underlying sucrose accumulation. Collectively, these results support the scientific rationale of using SSC as an effective surrogate for sugar accumulation to rapidly mine associated genes while balancing efficiency and cost [34].

**Figure 7:**
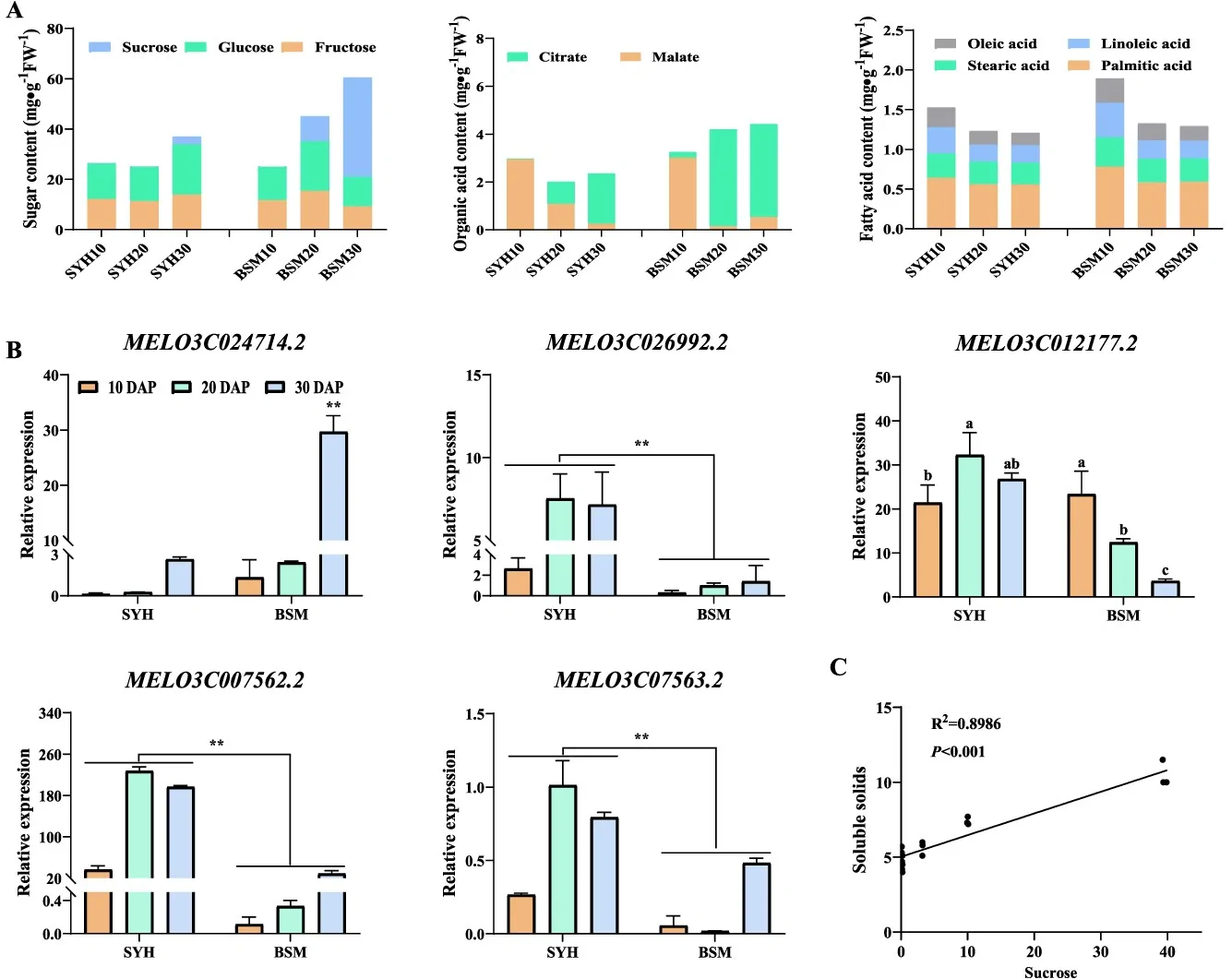
Metabolic profiling and candidate gene expression between SYH and BSM fruits. (A) Metabolic dynamics of soluble sugar, organic acid, and fatty acid content in SYH and BSM fruits at 10, 20, and 30 DAP. (B) Expression patterns of key candidate genes in SYH and BSM fruits at 10, 20, and 30 DAP. Bar plots represent the relative expression levels determined by qRT-PCR (means ± SD, *n* = 3). Asterisks indicate significant differences (^**^P < 0.01, ^*^P < 0.05; Student’s *t*-test). Lowercase letters represent significant differences at *P* < 0.05 (one-way ANOVA followed by Tukey’s HSD test). (C) Correlation analysis between SSC and sucrose content in SYH and BSM fruits.

To further validate the five key candidate genes identified in this study, we examined their expression patterns in SYH and BSM fruits at 10, 20, and 30 DAP using qRT-PCR (Fig. 7B). *MELO3C024714.2*, an ERF transcription factor, exhibited significantly higher expression in BSM at 30 DAP compared to other stages. Given that homologs of *MELO3C024714.2* function in sugar metabolism [29, 35–38], we investigated its potential role. Our phylogenetic analysis revealed that the protein encoded by *MELO3C024714.2* contains a AP2 superfamily domain and shares identical conserved motifs with CitERF16, with which it clusters in the same clade (Supplementary Fig. S3). We therefore propose that it acts as a positive regulator of sugar accumulation during fruit ripening. In contrast, *MELO3C026992.2*, encoding a sugar transport protein, showed higher expression in SYH than in BSM, contrary to the expectation for a positive regulator of sugar accumulation. Meanwhile, *MELO3C012177.2*, a bHLH transcription factor, displayed a biphasic trend in SYH (first increasing then decreasing), whereas its expression declined continuously in BSM. Given that bHLH transcription factors are known to regulate organic acid accumulation [39–41], the inverse correlation between *MELO3C012177.2* expression and organic acid levels in both accessions suggests a negative regulatory role for this gene. *MELO3C007562.2* and *MELO3C007563.2* are annotated as class 3 lipases involved in triglyceride degradation within the glycerolipid metabolism. Their expression levels were significantly higher in SYH compared to BSM, and both genes exhibited marked upregulation during the later developmental stage. In contrast, fatty acid content in both accessions peaked at 10 DAP and then declined sharply. This inverse temporal pattern suggests that *MELO3C007562.2* and *MELO3C007563.2* may act synergistically to negatively regulate fatty acid accumulation, potentially driving the rapid degradation observed after 10 DAP.

## Discussion

### The central role of SSC in melon fruit quality

Fruit quality has emerged as a major focus in modern horticulture, with sensory attributes—including aroma, appearance, taste, and nutritional value—driving contemporary breeding objectives. In terms of taste, SSC serves a fundamental and integrative indicator of fruit quality, reflecting the combined contributions of soluble sugars, organic acids, vitamins, and soluble proteins. Moreover, SSC is widely employed as a practical metric for assessing melon maturity and determining optimal harvest timing [33, 42, 43].

As an economically important annual vine crop within the Cucurbitaceae family and a globally significant horticultural commodity, melon is prized for its internal quality attributes, which strongly influence consumer preference [44]. Despite recent advances in genomics and phenomics have accelerated the identification of genes governing fruit quality, the genetic architecture underlying SSC, a key determinant of flavor in melon, remains incompletely explored.

In this study, we leveraged genomic data from a core collection of 200 diverse melon accessions coupled with multienvironment measurements to conduct a GWAS, identifying 79 significant SNPs associated with SSC. Integration of transcriptomic profiles from contrasting genotypes further prioritized 18 candidate genes, 14 of which were consistently validated by qRT-PCR as likely regulators of SSC in melon fruit. Haplotype analysis provided additional genetic support for the functional relevance of these loci in modulating SSC variation across the population.

### Reliability of key candidate genes identified through an integrated multiomics approach

Currently, research on the genetic regulation of SSC is relatively advanced in tomato. For instance, Zhang *et al.* [45] identified an SNP on chromosome 11 strongly associated with SSC and demonstrated that *SlCDPK27* negatively regulates sugar accumulation. Wang *et al.* [18, 45] characterized *SlSTP1*, encoding a sugar transporter protein, and demonstrated that binding of ZAT10-like to the *STP1^Insertion^* promoter reduces SSC in modern cultivars. Similarly, Li *et al.* [46] reported that the zinc finger protein C2H2-71 suppresses expression of the cell wall invertase *SlLIN5*, thereby negatively modulating SSC in red-ripe tomato fruit.

In cucurbits, Guo *et al.* [47] mapped two putative major-effect loci for SSC on watermelon linkage group 1, while Diaz *et al.* [14] identified QTL clusters associated with SSC on chromosomes 2, 3, and 5. In melon, Paris *et al.* [13] consistently detected two QTLs (*SSC7.4* and *SSC10.8*) related to SSC, and more recently, Yan *et al.* [26] identified 138 loci associated with SSC. At the present study, integration of GWAS and transcriptomic profiling enabled the identification of 14 high-confidence candidate genes associated with SSC, which located within 10 QTL intervals. Notably, five of these QTLs overlapped with previously reported genomic regions [12, 33] (Table 1). The candidate genes includes transcription factors from the ERF and bHLH families, as well as a sugar transporter (STP14-like), all of which have been implicated in sugar accumulation [29, 45, 48–50]. Furthermore, Zhao *et al.* [51] highlighted the role of glycosyltransferases in soluble sugars and organic acid metabolism. Consistent with this, the lead SNP chr02_21 790 857 within *qSSC2.4* resides in *MELO3C026990.2* (Supplementary Fig. S2), a gene homologous to glycosyltransferase. Adjacent to it, *MELO3C026992.2* was annotated as a sugar transporter protein, suggesting potential functional synergy in sugar metabolism.

Notably, the gene model for *MELO3C007562.2* is incomplete in the DHL92 (v3.6.1) reference genome. Alignment with the IVF77 reference genome revealed that the lead SNP chr08_3 682 159 lies within an intronic region of a lipase-encoding gene. Previous studies have reported that stored lipids are hydrolyzed, and subsequently converted to sucrose via glyoxylate cycle and gluconeogenesis during seed germination [52, 53], with lipases catalyzing the initial step of triacylglycerol breakdown [53–55]. Beyond energy mobilization, lipases also influence fruit flavor by modulating volatile compound biosynthesis [51]. Furthermore, *MELO3C012838.2* and *MELO3C011750.2*, identified as putative domestication-related genes, reside in genomic regions differentiated between *C. melo* ssp*. melo* and *C. melo* ssp*. agrestis* [23], supporting the hypothesis that SSC is a domestication-selected trait in melon. Collectively, these 14 key candidate genes provide valuable insights into the molecular underpinnings of melon fruit flavor quality.

### Metabolic trade-off: a model for LGG-mediated regulation of flavor quality in melon fruit

During plant growth and development, the LGG pathway functions as a central regulatory module that coordinates energy metabolism and cellular homeostasis. By integrating GWAS and RNA-seq analyses, we identified five key genes (*MELO3C024714.2, MELO3C026992.2*, *MELO3C012177.2*, *MELO3C007562.2*, and *MELO3C007563.2*) within this pathway, which showed differential expression between SYH and BSM and exhibited significant haplotype-phenotype associations. These findings provide a framework for understanding how lipid-derived carbon skeletons are partitioned between gluconeogenesis and alternative metabolic fates.

A significant decline in fatty acids was observed from 10 to 20 DAP in both SYH and BSM. However, concomitant sucrose accumulation occurred only in BSM, which is consistent with the classic ‘lipid degradation-gluconeogenesis’ carbon source supply model [*56**,*  *57*]. Notably, two key candidate genes, *MELO3C007562.2* and *MELO3C007563.2*, which encode lipases catalyzing the hydrolysis of triglycerides to release fatty acids, were highly correlated in the co-expression network (PCC = 0.874) [*58*]. These genes likely provide the carbon skeletons and energy source for β-oxidation, glyoxylate cycle, and gluconeogenesis within the lipid metabolism of the LGG pathway. However, despite similar rates of fatty acid degradation in SYH, sucrose synthesis was not triggered, and sucrose content remained low throughout the developmental period. This result indicates that while lipid degradation is a necessary condition for gluconeogenesis, it is not a sufficient one. Therefore, we propose that two complementary mechanisms—metabolic shunt and metabolic switch—collectively determine the genotype-specific carbon allocation.

Lipid-derived carbon appears to be shunted away from gluconeogenesis toward oxidative phosphorylation [*59**,*  *60*], membrane remodeling [*56**,*  *61**,*  *62*] and volatile organic compound (VOC) biosynthesis [*63*]. In this study, GO enrichment analysis revealed that DEGs were significantly overrepresented in generation of precursor metabolites and energy (GO:0006091), fatty acid β-oxidation (GO:0006635), and oxidoreductase activity (GO:0016491), which may underlie the sharp reduction in fatty acid content. Furthermore, KEGG enrichment analysis highlighted significant enrichment in glycerolipid metabolism (ko00561) and phosphatidylinositol signaling system (ko04070), indicating a tight coupling between membrane lipid remodeling and signal transduction. Specifically, *MELO3C015183.2* (encoding phosphatidylcholine 1-acylhydrolase [*58*]), which is mapped to the LGG pathway, exhibited higher expression in SYH, suggesting a potential mechanistic link to the divergence in lipid metabolite partitioning between the two accessions. Together, these pathways illustrate how SYH prioritizes energy production and membrane homeostasis over sugar storage.

The ‘metabolic switch’ governing gluconeogenic activity is differentially regulated between genotypes, likely existing in an off or suppressed state in SYH, whereas it functions normally in BSM. This differential regulation is orchestrated through several interconnected mechanisms. First, regulation of key enzyme expression. Previous study showed that the activity of phosphoenolpyruvate carboxykinase (PEPCK), a rate-limiting enzyme in gluconeogenesis, exhibits genotype-specific differences [*64*]. In the LGG pathway, transcript levels of the corresponding genes (*MELO3C018724.2*, *MELO3C025636.2*, and *MELO3C024646.2*) were significantly higher in BSM than in SYH, consistent with their established roles. Second, concerning the transcriptional cascade of key regulatory factors. In grape, VvERF045 acts with co-factors to enhance transcriptional activation of *VvSPS4* (sucrose phosphate synthase), thereby promoting sugar accumulation [*36*]. Similarly, *MELO3C024714.2* (an ERF transcription factor [*58*]) showed high expression in BSM but low expression in SYH. This pattern mirrors the aforementioned regulatory mechanism, suggesting its role as a positive regulator of gluconeogenesis in melon. Third, regarding the genotype-specific differentiation of transport functions. Sucrose is interconverted with glucose and fructose by sucrose synthase (SuSy) [*65**,*  *66*] and invertase (INV) [*67*], then transported and stored in the vacuole via sugar transporters [*68*]. Cytosolic hexose concentration mediates the feedback regulation of sucrose-degrading enzymes via sugar signaling pathways. In BSM, the low expression of *MELO3C026992.2* (encoding a monosaccharide transporter [*69*]) likely leads to elevated cytosolic hexose levels, which may repress the activity of sucrose-degrading enzymes (SuSy and INV) through sugar-mediated feedback, thereby attenuating sucrose hydrolysis and facilitating sucrose accumulation. Conversely, in SYH, high expression of this gene maintains low cytosolic hexose levels, allowing continued sucrose degradation and resulting in a hexose-dominated accumulation pattern. This interpretation is supported by findings that silencing *LeHTs* (hexose transporters) in tomato reduced hexose accumulation by 55% *[*68*]*. Similarly, this mechanism accounts for the 38% decrease in hexose content observed in BSM. Additionally, we developed a molecular marker based on *MELO3C026992.2* for predicting SSC in melon fruits (Supplementary Fig. S4). This marker enables early quality assessment, significantly shortens the breeding cycle, and facilitates commercial variety selection.

Organic acid metabolism, a core component of the LGG pathway, is intricately coupled with transcriptional regulation. Previous weighted gene co-expression network analysis has identified bHLH transcription factors as key regulators of organic acid accumulation, linking them to multiple signaling pathways through their co-expression patterns [*70*]. This aligns with functional studies in apple and citrus that established the role of bHLH family members in organic acid metabolism [*39**,*  *40*]. In this study, the expression of *MELO3C012177.2* (a bHLH transcription factor [*58*]) exhibited an inverse relationship with organic acid accumulation, suggesting a negative regulatory role that corroborates previously characterized functions [*39**,*  *41*]. Given that organic acids originate from the glyoxylate cycle, this gene likely serves as a nodal factor bridging lipid signaling and carbon partitioning.

This study redefines the biological significance of lipid degradation across different genetic backgrounds. In BSM (high SSC), lipid degradation is temporally coupled with sucrose accumulation, channeling lipid-derived carbon toward gluconeogenesis—a pattern described as ‘fueling sugar accumulation’, consistent with the classical model. Conversely, in SYH (low-SSC), lipid-derived carbon is diverted toward nonsugar metabolic pathways, representing a ‘fueling homeostatic maintenance’ strategy. We propose that this genotype-specific carbon partitioning is governed by two complementary mechanisms: the metabolic shunt, determining the alternative fates of lipid-derived carbon, and the metabolic switch, regulating the activation of gluconeogenic activity downstream of lipid degradation. This integrated model provides a new perspective on the metabolic basis of fruit quality formation and offers a theoretical foundation for optimizing carbon partitioning in melon breeding.

In summary, SSC regulation in melon is governed by a dynamic interplay of genetic, developmental, and environmental factors. While our integrative multiomics approach has identified 14 high-priority candidate genes, their precise contributions to sugar accumulation, acid metabolism, and overall flavor require functional validation. Future work should prioritize stable genetic transformation [71–73], elucidation of transcriptional and metabolic regulatory networks, and evolutionary analyses of domestication signatures to clarify how these genes orchestrate soluble sugars, acids, and other quality determinants. Such efforts will not only advance our fundamental understanding of fruit quality regulation but also accelerate molecular breeding for superior melon varieties with enhanced flavor and market appeal.

## Materials and methods

### Plant materials and growth conditions

A total of 200 melon accessions collected worldwide were used for the GWAS, including 98 thin-skinned, 66 thick-skinned, and 36 wild accessions. All plants were cultivated in plastic greenhouses at the Maozhuang Research Station of Henan Agricultural University (Zhengzhou, China), during the spring and autumn seasons of 2017, 2018, and 2023. For each accession, at least three individual plants were grown as biological replicates, and one fruit per plant was harvested for phenotypic analysis. Fruits were harvested uniformly at 30–45 DAP.

For RNA-Seq analysis, SYH (low SSC) and BSM (high SSC) were selected as experimental materials. These plants were grown at the same research station during the spring of 2025. Each sample included three biological replicates (individual plants), with one fruit retained per plant. Fruit flesh samples were collected at 10, 20, and 30 DAP for subsequent transcriptomic profiling.

### Measurement of fruit quality traits and data processing

The SSC of fruit flesh was measured using a handheld refractometer (expressed in °Brix). For each mature fruit, juice was extracted from the equatorial center region using gauze. Soluble sugars (glucose, fructose, and sucrose) and organic acids (citrate, and malate) were quantified using high-performance liquid chromatography, while fatty acid composition (palmitic acid, stearic acid, linoleic acid, and oleic acid) was analyzed by gas chromatography–mass spectrometry (GC–MS) at Nanjing Webiolotech Testing Technology Co., Ltd. Three technical replicates were performed per sample, and their mean value was recorded as one biological replicate. Phenotypic data for each melon accession in each environment were represented by the mean of at least three biological replicates. After SSC measurement, flesh samples were immediately frozen in liquid nitrogen and stored at −80°C for further analysis. The R package lme4 v1.1-35.5 was employed to fit the phenotypic data across six environments into Best Linear Unbiased Prediction (Blup) and to calculate broad-sense heritability (*H^2^*). The phenotypic data from the six environments, along with the Blup values, were used to construct an association mapping panel.

### Genome-wide association study

Genomic data for the 200 melon accessions were obtained from the NCBI SRA database (https://www.ncbi.nlm.nih.gov/sra) under accession number PRJNA565356. All paired-end sequencing reads were aligned to the melon reference genome (DHL92 v3.6.1) using GATK v4.0 (http://www.broadinstitute.org/gatk). To prepare the phenotype–genotype data for subsequent association analysis, a high-density genotyping subset of 2 538 320 SNPs was constructed by filtering all SNPs with a minor allele frequency > 0.05 and a missing rate < 20%.

GWAS was conducted using both the MLM and the CMLM in TASSEL_v5 (https://bitbucket.org/tasseladmin/tassel-5-standalone/downloads/?tab=tags) to assess the associations between SNP loci and the SSC trait. The first five principal components, calculated using LDAK (https://github.com/dougspeed/LDAK/), were incorporated into both models to account for population structure, and a genetic kinship matrix was calculated. The level of LD was assessed using the software PopLDdecay (https://github.com/BGI-shenzhen/PopLDdecay) by calculating the physical distance at which the r^2^ value dropped to half its maximum. The estimated LD decay distance was 94.5 kb (Supplementary Fig. S5). The genome-wide significance threshold was set to 1/n (where n represents the number of effective independent SNPs), corresponding to *P* < 3.94 × 10^−7^, to define significantly associated SNP loci. Manhattan plots were generated using the R packages ggplot2 v3.5.1 and CMplot v4.5.1.

### Identification of candidate genes and haplotype analysis

All significant SNPs (*P* < 3.94 × 10^−7^) identified from MLM and CMLM models across the six environments and the Blup dataset were integrated to identify potential candidate genes associated with SSC in melon fruit. The physical positions of the SNPs were determined based on the melon reference genomes DHL92 v3.6.1 and IVF77 v1. The annotation information of candidate genes was obtained from the DHL92 reference genome. LDBlockShow was used to visualize linkage disequilibrium and construct haplotype blocks [74]. Genes located within these LD blocks containing lead SNPs were considered as potential candidate genes.

Genomic variations, including gene full length sequences and promoter regions (2 kb upstream of the transcription start site) within the natural melon populations, were annotated using snpEff (https://pcingola.github.io/SnpEff)*.* Subsequently, major variations, such as exonic SNPs causing nonsynonymous mutations, SNP leading to alternative splicing, and SNPs in promoters, were extracted for haplotype analysis using geneHapR v1.2.4.

### RNA-Seq and differential expression analysis

All flesh samples were sent to Nanjing Genepioneer Biotechnology for RNA-Seq. The total RNA was extracted using a universal RNA extraction kit (R401, Vazyme Biotech, China). The libraries were constructed for transcriptome sequencing following quality control verification, and paired-end sequencing was performed on the Illumina NovaSeq 6000 platform. Raw data were processed with fastp (https://github.com/OpenGene/fastp) to obtain clean data, which were then aligned to the DHL92 reference genome using BWA v0.7.17. PCA was performed using the prcomp function in R and visualized with the factoextra v1.0.7. Transcripts Per Million (TPM) were calculated to assess gene expression abundance and variation by setdiff. Heatmap drawn by TBTools [75]. Differential expression analysis of genes was carried out using DESeq2 v1.40.2, with thresholds of adjusted *P*-value (padj) < 0.05 and absolute log_2_ fold change |log_2_FC| > 1 to identify significantly DEGs. Subsequently, DEGs were subjected to GO and Kyoto Encyclopedia of Genes and Genomes (KEGG) enrichment analyses to elucidate potential biological functions and regulatory networks. The raw transcriptome data have been deposited in the NCBI BioProject database under the accession number PRJNA1392061.

### qRT-PCR validation

Total RNA was extracted using a plant RNA kit and reverse-transcribed using reverse transcriptase (RC411 and R333, Vazyme Biotech, China). The melon actin gene (*MELO3C008032.2*) was used as the internal reference gene^[^^76^^]^. qRT-PCR was carried out using 2 × Universal Blue SYBR Green qPCR Master Mix (Servicebio, China) on a CFX96 Real-Time System (Bio-Rad, USA). All reactions were conducted with three biological replicates and three technical replicates. The relative expression levels of candidate genes were calculated using the 2^–ΔΔCt^ method [77, 78]. The sequences of the primers for qRT-PCR are listed in Supplementary Table S8.

### Development of cleaved amplified polymorphic sequence marker

Following the method described previously [79], a CAPS marker was developed based on the G/C SNP of the *MELO3C026992.2* gene, which correlates high- and low-SSC in melon fruits. PCR amplification was performed using genomic DNA extracted from 14 melon germplasm resources. The resulting PCR products were digested with the *Taq*I restriction endonuclease and separated by native polyacrylamide gel electrophoresis. Detailed primer information is provided in Supplementary Table S8.

### Phylogenetic analysis

Protein sequences of ERF transcription factors involved in fruit sugar accumulation were retrieved from the NCBI GenBank database based on the reported accession numbers. Multiple sequence alignment was performed using MEGA12 with the MUSCLE algorithm, and a phylogenetic tree was constructed using the neighbor-joining (NJ) method with 1000 bootstrap replicates. Conserved motifs were identified using the MEME Suite, and conserved domains were analyzed via the NCBI CDD Batch Search. The phylogenetic tree, motif compositions, and domain architectures were integrated and visualized using TBtools [75].

## Supplementary Material

Web_Material_uhag196

## Data Availability

The raw data of RNA-seq are deposited in the National Center for Biotechnology Information with BioProject: PRJNA1392061. The datasets supporting the findings in this study are available in the supplementary materials.
