# Supplementary material for: Integrating genome-wide association study and transcriptomic profiling identifies candidate genes underlying soluble solids content in melon (Cucumis melo L.)
Source: Hortic Res. 2026 May 13;13(10):uhag196. doi: 10.1093/hr/uhag196 (PMC13630446; doi:10.1093/hr/uhag196)
Supplement: Supplementary file 1 [file web_material_uhag196.zip › Supplement Figure-SSC-20260331.docx]

**Supplementary Fig. S1.** Expression validation of four candidate genes between SYH and BSM fruits at 20 DAP. Left and right Y-axis represent qRT-PCR and RNA-Seq data, respectively. Bar plots represent the relative expression levels determined by qRT-PCR (means ± SD, *n* = 3). Asterisks indicate significant differences (***P*<0.01, **P*<0.05; Student’s *t*-test). Superimposed lines with dots represent the TPM values obtained from RNA-Seq data.

**Supplementary Fig. S2**. Nonsynonymous mutation and haplotype analysis of key candidate genes associated SSC. (A-I) Gene structures and haplotype effects for (A) *MELO3C011750.2*, (B) *MELO3C012838.2*, (C) *MELO3C012177.2*, (D) *MELO3C007563.2,* (E) *MELO3C008705.2*, (F) *MELO3C022401.2*, (G) *MELO3C026992.2*, (H) *MELO3C022400.2*, (I) *MELO3C029714.2*, and (J) *MELO3C024714.2*. Green boxes represent exons, lines represent introns, orange boxes represent the 5'UTR and 3'UTR, arrows indicate transcriptional direction. Bar graphs show haplotype-associated phenotypic differences in SSC. Lowercase letters indicate statistically significant differences at *P* < 0.05 (one-way ANOVA followed by Tukey's HSD test). **, *P* < 0.01 (Student's *t*-test).

**Supplementary Fig. S3.** Phylogenetic relationships, conserved motifs, and functional domains of sugar metabolism-related ERF transcription factors. (A) Phylogenetic tree of ERF transcription factors. The tree was constructed using the neighbor-joining (NJ) method with 1,000 bootstrap replicates. The scale bar represents the number of amino acid substitutions per site. (B) Conserved motif compositions. Ten distinct motifs (Motif 1–10) were identified using the MEME Suite. Different motifs are represented by colored boxes as indicated in the legend, with motif numbers labeled on the right. (C) Conserved domain architectures. Domains were identified using the NCBI Conserved Domain Database (CDD) batch search.

**Supplementary Fig. S4**. Validation of the efficiency of CAPS marker in different melon accessions (7 high SSC accessions and 7 low SSC accessions).

**Supplementary Fig. S5.** Genome-wide average linkage disequilibrium (LD) decay estimated from different melon accessions.
